# Green fabrication of Co and Co_3_O_4_ nanoparticles and their biomedical applications: A review

**DOI:** 10.1515/biol-2021-0003

**Published:** 2021-01-20

**Authors:** Abdul Waris, Misbahud Din, Asmat Ali, Shakeeb Afridi, Abdul Baset, Atta Ullah Khan, Muhammad Ali

**Affiliations:** Department of Biotechnology, Quaid-i-Azam University, Islamabad, Pakistan; Centre for Human Genetics, Hazara University Mansehra, Pakistan; Department of Zoology, Bacha Khan University Charsadda, Pakistan; Department of Biotechnology, University of Malakand, Chakdara Dir Lower, Pakistan

**Keywords:** nanotechnology, green synthesis, cobalt nanoparticles, cobalt oxide nanoparticles, biomedical applications of nanoparticles

## Abstract

Nanotechnology is the fabrication, characterization, and potential application of various materials at the nanoscale. Over the past few decades, nanomaterials have attracted researchers from different fields because of their high surface-to-volume ratio and other unique and remarkable properties. Cobalt and cobalt oxide nanoparticles (NPs) have various biomedical applications because of their distinctive antioxidant, antimicrobial, antifungal, anticancer, larvicidal, antileishmanial, anticholinergic, wound healing, and antidiabetic properties. In addition to biomedical applications, cobalt and cobalt oxide NPs have been widely used in lithium-ion batteries, pigments and dyes, electronic thin film, capacitors, gas sensors, heterogeneous catalysis, and for environmental remediation purposes. Different chemical and physical approaches have been used to synthesize cobalt and cobalt oxide NPs; however, these methods could be associated with eco-toxicity, cost-effectiveness, high energy, and time consumption. Recently, an eco-friendly, safe, easy, and simple method has been developed by researchers, which uses biotic resources such as plant extract, microorganisms, algae, and other biomolecules such as starch and gelatin. Such biogenic cobalt and cobalt oxide NPs offer more advantages over other physicochemically synthesized methods. In this review, we have summarized the recent literature for the understanding of green synthesis of cobalt and cobalt oxide NPs, their characterization, and various biomedical applications.

## Graphical abstract

**Figure:**
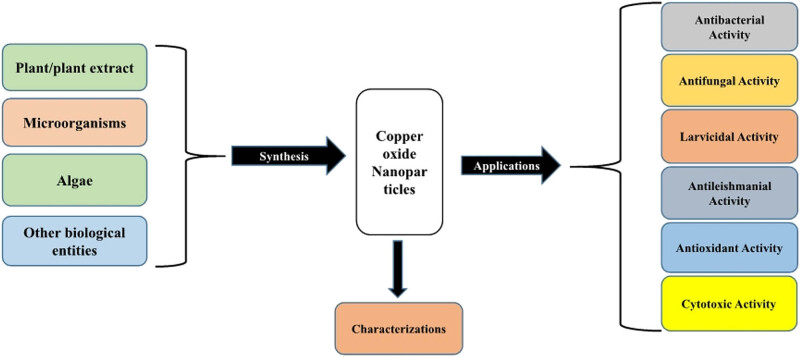

## 1 Introduction

Nanotechnology combines various chemical and physical processes to construct nanomaterials, which are less than 100 nm in at least one dimension, and have unique properties [1]. Nanotechnology has applications across different fields, such as nanomedicines, biomaterials, nanoelectronics, environment, imaging, industries, and agriculture [2,3]. In healthcare, it has been widely used for the diagnosis and treatment of diseases, drug delivery, and novel drug formulations [2,4].

Cobalt is a transition metal that has a beneficial effect on human health [5,6]. It constitutes a part of vitamin B12, which is useful in anemia treatment as it provokes the formation of red blood cells [6]. Cobalt has unique magnetic, optical, electrical, and catalytic characteristics that make it suitable for a wide range of applications in the field of nanoelectronics and nanosensors [7,8,9]. Cobalt can exhibit variable oxidation states (Co^2+^, Co^3+^, and Co^4+^), which makes it attractive to be used in several industries [10]. Because of this multivalent state, cobalt has the ability to be present in different spin states in its oxide forms, i.e., low, intermediate, and high [10,11].

Recently, cobalt nanoparticles (CoNPs) have attracted considerable attention because they are more economical than the noble metal nanoparticle (NP) and show different properties, such as electrical and magnetic, due to their large surface area [12,13]. CoNPs have been explored as a therapeutic agent for the treatment of diseases, such as microbial infection, which make them attractive for biomedical applications [14,15]. CoNPs are nontoxic in the body at lower levels, have strong activities against bacteria and fungi at lower concentrations, and have fewer side effects than antibiotics [16,17].

**Figure 1:**
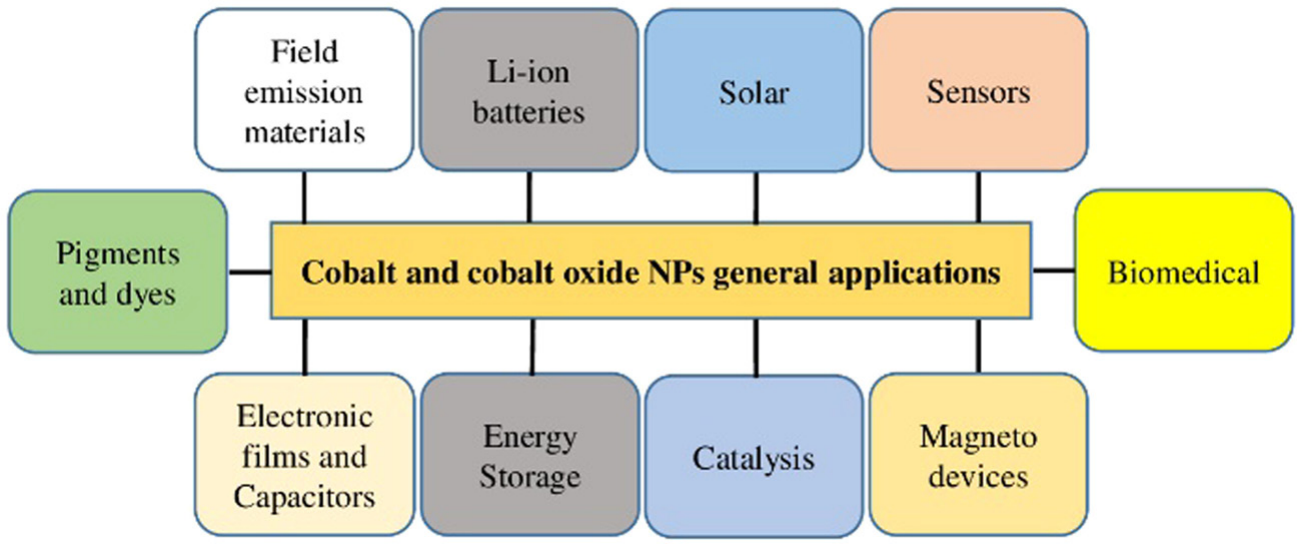
General applications of cobalt and cobalt oxide NPs.

Different types of NPs and their various applications in the area of medicine, textiles, cosmetics, electronics, optics, energy generation, and environmental science have been reported [18,19,20,21,22]. These NPs include silver NPs (AgNPs), iron NPs, CoNPs, copper NPs, gold NPs, silica NPs, platinum NPs, palladium NPs, zinc oxide NPs, magnesium oxide NPs, cerium dioxide NPs, and titanium oxide NPs [18]. Among all NPs, cobalt and cobalt oxide (Co_3_O_4_) NPs have been exploited the most because of their unique and wide range of applications [5,6]. Co_3_O_4_ is an antiferromagnetic p-type semiconductor with a direct optical band gap of 1.48 and 2.19 eV [23,24]. Co_3_O_4_ is a multifunctional material and has many applications such as biomedical applications (antibacterial, antiviral, antifungal, antileishmanial, therapeutic agents, anticancer, and drug delivery), gas sensors, solar selective absorbers, anode materials in lithium-ion batteries, energy storage, pigments and dyes, field emission materials, capacitors, heterogeneous catalysis, magneto-resistive devices, and electronic thin films [25,26,27,28,29] as shown in Figure 1. The oxides of cobalt are abundant in nature, as only the Co_3_O_4_ and CoO are stable [30], with Co_3_O_4_ possessing the highest stability. In this review, we aim to focus on the biological synthesis, characterization, and biological activities of cobalt and cobalt oxide NPs.

## 2 Synthesis of NPs

**Figure 2:**
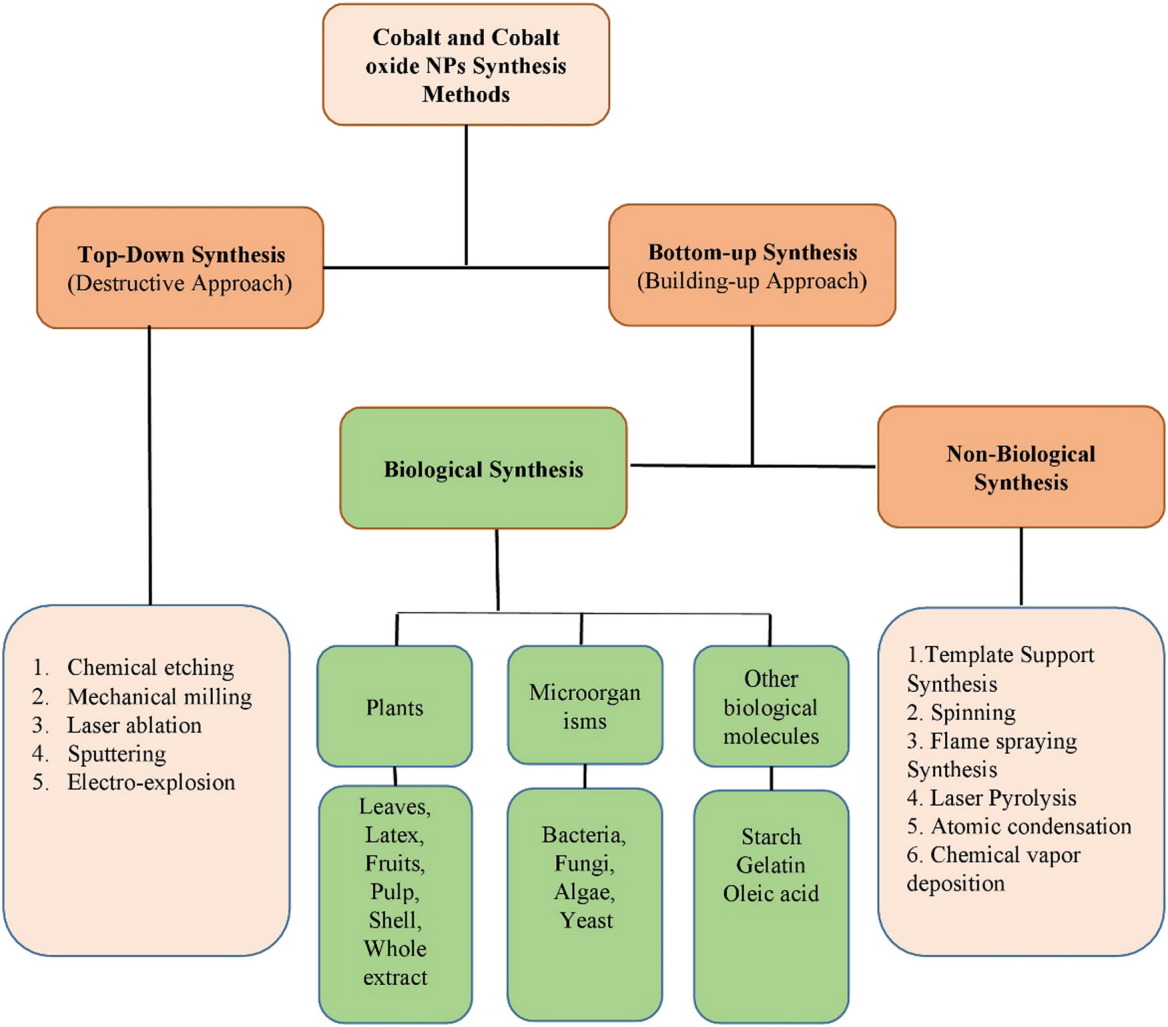
Various methods for the synthesis of cobalt and cobalt oxide NPs.

Different approaches can be used for the synthesis of NPs. These approaches are divided into two classes: top-down approach and bottom-up approach. A top-down approach is a destructive approach in which larger molecules are decomposed into smaller ones, and these smaller molecules are then converted into suitable NPs, whereas the bottom-up is a building approach that involves the assembly of atomic size to form nano-size particles [1]. Examples of the top-down approach include chemical etching, laser ablation, mechanical milling, electro-explosion, and sputtering. In these different methods, the bulk materials are first converted into powder form and then into particular NPs. Each synthesis method has its advantages and limitations. The top-down approach has many advantages, such as the production of the desired size and large quantity of nanoparticle, whereas the fabrication of NPs using this method leads to eco-toxicity, high energy consumption and on top of that is expensive and time-consuming [2,3]. The bottom-up approach is further divided into two classes: biological and non-biological approaches. Examples of non-biological approaches include template support synthesis, flame spraying, spinning, laser pyrolysis, atomic condensation, and deposition of chemical vapors. These methods also use toxic chemicals, are expensive and time-consuming, whereas the biological approach uses various biotic resources such as plants, algae, microorganisms, and other biological molecules like starch, egg albumin, and gelatin for the production of different types of NPs. This biological approach is also known as the green approach [2,3,4,5,6,7,8,9,10]. This method is eco-friendly, simple, reliable, biocompatible, and easy for the synthesis of NPs. The classification of various methods of fabrication of NPs is shown in Figure 2. However, in this review, we only focus on the green synthesis of cobalt and cobalt oxide NPs, and their biological applications.

## 3 Green synthesis of cobalt and cobalt oxide NPs (biological methods)

**Figure 3:**
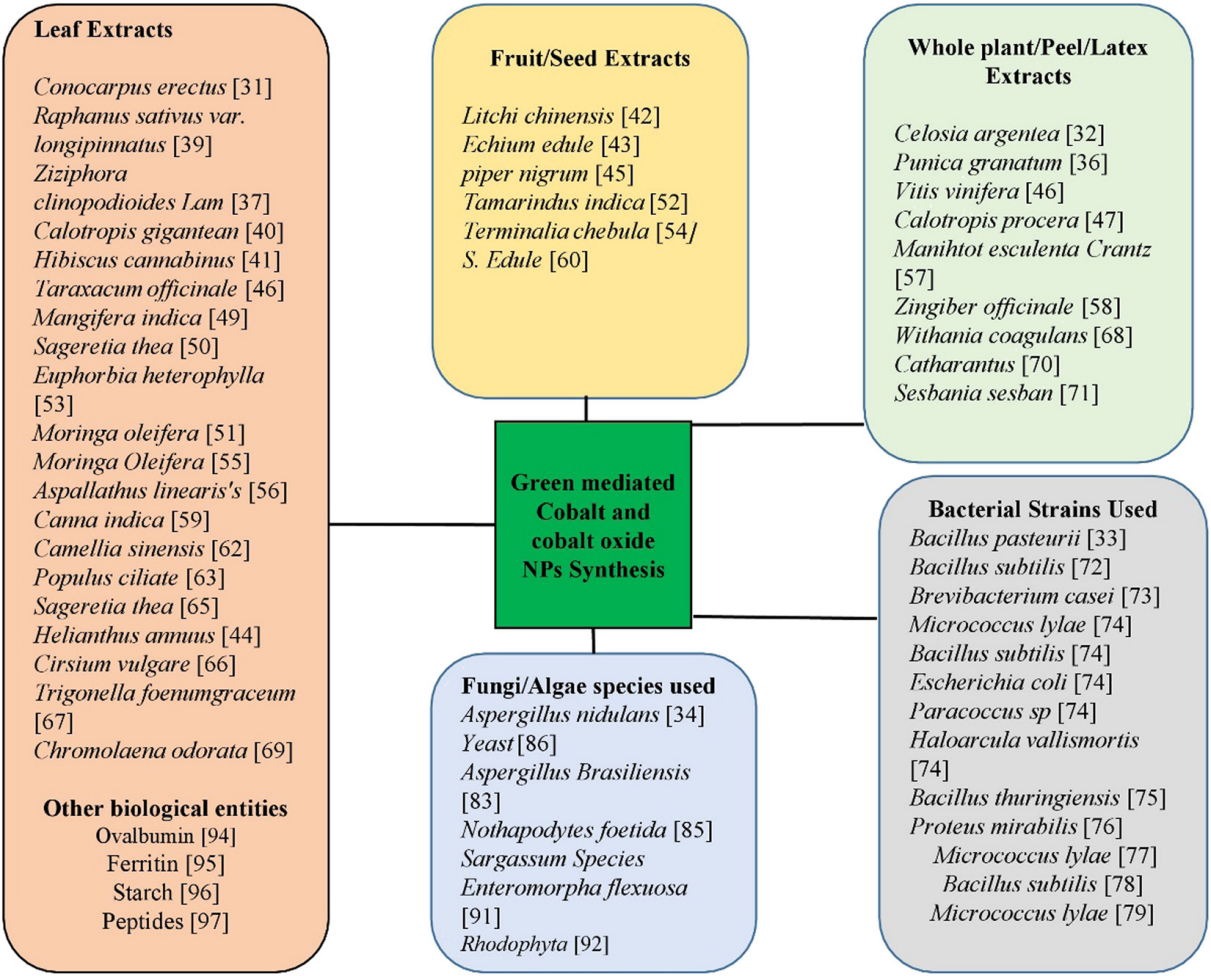
Use of different plant/parts of plant, microorganism, and other biological molecules for the synthesis of cobalt and cobalt oxide NPs.

The synthesis of NPs by physical and chemical methods (traditional approaches) has some detrimental effects such as emission of highly expensive and toxic chemicals that carry many threats to the ecosystem, as well as require high energy consumptions, high cost, and time-consuming processes. To overcome these problems, green synthesis of NPs is being implemented. Green-mediated approach emerged as an eco-friendly, biocompatible, and most fascinating, offering more advantages as compared to other traditional approaches [31,32]. In the green-mediated synthesis of NPs, different plants/parts of plants, fruits, bacteria, algae, fungi, and other biological molecules such as starch and egg albumin have been used as a reducing/capping/oxidizing agent [33,34,35]. Different parts of plants, microorganisms, and other biological molecules have been exploited for the fabrication of cobalt and cobalt oxide NPs as shown in Figure 3. These biological resources contain different biomolecules and metabolites that are responsible for the oxidation/reduction, stabilization, and production of particular NPs. Cobalt and cobalt oxide NPs have been synthesized using different methods such as chemical, physical, and biological methods. However, biological approach resulted in less contaminated, safer, cost-effective, and large scale production of NPs [6,31,32].

### 3.1 Green synthesis using plant extracts

In contrast to bacteria, algae, and fungi, plants have been extensively used to synthesize cobalt and cobalt oxide NPs. This is because of their abundance, safe nature, and also the greater stabilization and reduction of plant phytochemicals. This method has been considered as an alternative to complex and costly physicochemical processes because of the following properties: viability, commercial feasibility, eco-friendliness, reliability, no waste generation, and simplicity [36,37]. Different parts of the plant such as leaves, roots, stem, fruits, seeds, latex, inner parts of the plant, shells, and peels have been used to synthesize Co and Co_3_O_4_ NPs as shown in Figure 3. The plant extracts have a variety of flavonoids, polysaccharides, amino acids, polyphenols, phenolic acids, ferulic acid, gentisic acid, terpenoids, thymol, tryptophan, and alkaloids, which act as a stabilizing, reducing, and chelating agent as presented in Table 1. These metabolites act as oxygen quenchers, reducing and stabilizing agents, metal chelating agents, and hydrogen donors. The reduction of metal ions by these substances within the plant extracts leads to the formation of respective nanomaterials [38]. Because of a rich source of metabolites, leaf extracts have been widely used for the synthesis of CoNPs. Plant-mediated synthesis of Co and Co_3_O_4_ NPs is a simple approach and there is no need for special requirements [6]. The metal salts have been mixed with the whole plant/part of plant extracts, and the reaction completes in a few hours under normal lab conditions [33,34,35,36,37,38].

**Table 1:** Plant-mediated synthesis of Co and Co_3_O_4_ NPs

Plant scientific name/common name	Part	NPs	Characterization	Phytoconstituents present in plant	Size (nm)	Shape	References
*Conocarpus erectus*/buttonwood or button mangrove	Leaf extract	Cobalt	SEM and XRD	Tannins, flavonoids, and phenolic acids	20–60	Spherical	[31]
*Celosia argentea*/plumed cockscomb or Silver cock’s comb	Whole plant extract	Cobalt	XRD, SEM, and EDX	Flavonoids, tannins, and phenolic acids	27.42		[32]
*Punica granatum*/pomegranate	Peel extract	Cobalt oxide	XRD, SEM, EDX, AFM, FTIR, and UV	Gallic acid, puni87 calagins A and B, ellagic acid, and gallotannins	40–80	Spherical	[36]
*Ziziphora clinopodioides* Lam/kakuti-e kuhi	Leaf extract	Cobalt	UV-vis, XRD, EDS, SEM, TEM, and FTIR	Flavonoids, α and β pinen, terpenoids, thymol, piperitenone, sis-isopulegone, pulegone, and cineol	28.19	Crystal	[37]
*Raphanus sativus* var. *longipinnatus/*Radish	Leaf extract	Cobalt	UV-vis, FTIR, SEM, and EDX	Ferulic acid, gentisic acid, raphanusin, erucic acid, sinapate, raphanin, and sulforaphen	80	Spherical	[39]
*Calotropis gigantea/*giant milkweed, crown flower, giant calotrope, swallow-wort	Leaf extract	Cobalt oxide	XRD, UV-vis, SEM, TEM, and EDX	Triterpenoids, flavonoids (polyphenols), steroids, cardenolides, and alkaloids	50	Spherical	[40]
*Hibiscus cannabinus/*Deccan hemp and Java jute	Leaf extract	Cobalt	XUV-vis, XRD, SEM, TEM, and FTIR	Phytosterols, flavonoids, polyphenols, tannins, steroids, alkaloids, saponins, lignans, essential oils, and glucosides	20.88	Crystalline	[41]
*Litchi chinensis/*litchi fruits	Fruits extract	Cobalt oxide	XRD, SEM, TEM, and FTIR	Phenolic acid, flavonoids condensed tannins, luteolin, anthocyanin, and proanthocyanidins.	NA	Rod like	[42]
*Sechium edule*/perennial climber	Fruit extract	Cobalt oxide	XRD, FTIR, TEM, AFM, SEM, and VSM	Ascorbic acid	3.79	Irregular	[43]
*Helianthus annuus*/sunflower	Leaf extract	Cobalt oxide	XRD, TGA, SEM	NA	NA	Plate	[44]
*Piper nigrum*	Seeds	Cobalt oxide	UV-vis, AFM, FTIR	NA	30–60	Spongy triangular	[45]
*Vitis vinifera/*common grape vine	Whole plant extract	Cobalt oxide	XRD, FT-IR, Raman, TEM, SAED, EDX, DRS, PL, and VSM	Phenolic, stilbenoids, anthocyanin, and acetylated anthocyanin	10–20	Rod shape	[46]
*Calotropis procera/*Sodom apple	Latex	Cobalt oxide	XRD, DSC, TEM, EDX, FTIR, and UV-vis	Tryptophan, alkaloids, resins, tannins calotropin, calactin, and calotoxin	10	Spherical	[47]
*Taraxacum officinale*/common dandelion	Leaf extract	Cobalt oxide	UV-vis, FT-IR, SEM, and TEM	Flavonoids and phenolic	50–100	Spherical	[48]
*Mangifera indica*/mango	Leaf extract	Cobalt	UV-vis, XRD, FT-IR, and SEM	Polyphenolics, flavonoids, and triterpenoids	25–40	Irregular shape	[49]
*Sageretia thea*/Osbeck.	Leaf extracts	Cobalt oxide	XRD, ATR-FTIR, HR-SEM, HR-TEM, SAED, and EDS	Friedeline, syringic acid, beta-sitosterol, daucosterol, gluco-syringic acid, and taraxerol	20.03		[50]
*Moringa oleifera*/drumstick tree, horseradish tree, and ben oil tree or benzolive tree	Leaf extract	Cobalt oxide	HR-TEM, EDS, FTIR, and AXS	α-Maltose, adenosine, catechin, chlorogenic acid, rutin, quercetin, kaempferol, caffeic acid, etc.	20–50	Symmetric	[51]
*Tamarindus indica/*tamarind fruit	Fruit pulp	Cobalt aluminate	FTIR, UV-vis, XRD, SEM, and RS	Tartaric acid, malic acid, and amino acids	71	Rectangular like	[52]
*Euphorbia heterophylla*/fire plant, painted euphorbia, Japanese poinsettia, desert poinsettia, and wild poinsettia	Leaf extract	Cobalt oxide	FTIR, XRD, Malvern Zetasizer Particle Size Analyzer, TEM, and UV-vis DRS	Alkaloid and saponin	69.75	Spherical	[53]
*Terminalia chebula/*black- or chebulic myrobalan	Fruit	Cobalt oxide	ATR-FTIR, XRD, TEM, SEM, and EDS	Hydrolysable tannins, gallic acid, chebulic acid, chebulic ellagitannins, and gallate esters	15–25	Spherical	[54]
*M. oleifera*/drumstick	Leaf extract	Cobalt	SEM, FTIR, UV-vis, and EDX	Oleic acid, ascorbic acid, dihexadecanoate, octadecenoic acid, methyl ester-hexadecanoic acid, and octadecenamide	168–295	Crystal	[55]
*Aspalathus linearis/*Rooibos	Leaf powder	Cobalt oxide	HR-TEM, EDS, Max solid-state Silicon drift detector, XRD, XPS, FTIR, and Raman spectroscopy	Phenolic compounds, aspalalinin, flavones, and flavonols	3.6	Quasi-spherical	[56]
*Manihtot esculenta Crantz*/cassava	Whole extract	Cobalt oxide	SEM, EDS, TEM, FT-IR, XRD, VSM, TGA, and EDX	Malonaldehyde, superoxide dismutase, reduced glutathione, and catalase	N/A	Prism like-anchored octahedron	[57]
*Zingiber officinale/*ginger	Whole plant	Cobalt	XRD, SEM, FTIR, VSM, and EDS	Gingerol, zingerone, shagaols, paradole, and starch	20–50	Crystal	[58]
*Canna indica/*Indian shot, African arrowroot, edible canna	Leaf extract	Bimetallic cobalt	UV-vis, TEM, EDX, and Thermo Electron LED	Glycosides, alkaloids, and terpenoids	450–550	Polydispersed	[59]
*S. edule/*chayote	Fruit extract	Bimetallic cobalt	AAS, SEM, SAED, TEM, XRD, and EPR	Trans-cinnamic acid, phenylacetic acid, trilinolenin, etc.	47.3	Prismoidal	[60]
*Cinnamomum verum/*true cinnamon tree or Ceylon cinnamon tree	Bark	Cobalt aluminate	MCM, XRD, SEM, TEM, XPS, IR, and UV-vis	Polysaccharides, polyphenols, flavonoids, and amino acids	50–60	Polyhedral	[61]
*Camellia sinensis*/tea plant, tea shrub, and tea tree	Leaf extract	Cobalt oxide	XRD, FESEM, EDX, HR-TEM, PL, FTIR, and UV-visible	Catechins, alkaloids, flavonoids, proteins, enzymes, vitamins, carbohydrates, polyphenols, lipids, and minerals	39.13	Quasi-rectangular	[62]
*Populus ciliata*/safaida	Leaf extract	Cobalt oxide	FTIR, TEM, SEM, and XRD	Alcohol-benzene, lignin, holocellulose, and alphacellulose	15–35	Square shape	[63]
*Tamarindus indica*/Indian tamarind	Fruit extract	Cobalt aluminate	FTIR, UV-vis, XRD, SEM, and TEM	Tartaric acid, malic and citric acid, potassium ditartrate, amino acids, and vitamin B	71.3	Cubic spinel	[64]
*S. thea*/Osbeck.	Leaf extract	Cobalt oxide	XRD, TEM, SEM, SAED, and EDS	Acids/base	20.03	Cubic	[65]
*Cirsium vulgare*	Leaf extract	Cobalt oxide	XRD, SEM, and TEM	NA	20		[66]
*Trigonella foenumgraceum/*fenugreek	Leaf extract	Cobalt oxide	FTIR, XPS, XRD, EDS, UV-vis, and TEM	Flavonoids, polyphenols, and different glycosides	13.2	Quasi-spherical	[67]
*Withania coagulans*	Whole plant extract	Cobalt oxide	UV-vis and XRD	Withanolide, withaferin, and withacoagin	NA	Cube shape	[68]
*Chromolaena odorata*	Leaf extract	Cobalt	UV-vis, XRD, FT-IR, and SEM	NA	20–49	Irregular, cubic, and hexagonal shapes	[69]
*Catharanthus roseus/*periwinkle	Whole plant extract	Cobalt	XRD, FESEM, and EDX	Alkaloids, tannins, flavonoids, polyphenols, and carbohydrates	27.08	Spherical shape	[70]
*Sesbania sesban*	Whole extract	Cobalt oxide	HR-TEM, EDAX, and XRD	NA	15–30	Spherical	[71]

Abbreviations: NPs, nanoparticles; NA, not available; SAED, selected area electron diffraction; UV-vis, ultraviolet visible spectroscopy; XPS, X-ray photoelectron spectroscopy; TEM, transmission electron microscopy; XRD, X-ray diffraction; AFM, atomic force microscopy; HR-SEM, high-resolution scanning electron microscopy; HR-TEM, high-resolution transmission electron microscopy; EDX, energy-dispersive X-ray(spectroscopy); FTIR, Fourier-transform infrared spectroscopy; NA, not available; UV-DRS, ultraviolet differential reflectance spectroscopy; TGA, thermo gravimetric analysis; VSM, vibrating sample magnetometer; PL, photoluminescence spectroscopy; PDXL, integrated X-ray powder diffraction software; EDS, energy-dispersive X-ray spectroscopy.

*Raphanus sativus* var. longipinnatus leaf extract was used to fabricate CoNPs with a spherical structure that represents the presence of ferulic acid, gentisic acid, raphanusin, erucic acid, sinapate, and sulforaphen. The green-synthesized NPs were characterized by UV-visible spectroscopy (UV-vis), X-ray diffraction (XRD), and scanning electron microscopy-energy-dispersive X-ray (spectroscopy) (SEM-EDX). These CoNPs showed potential antibacterial activities and also showed promising cytotoxic activities against the HeLa cancer cell lines *in vitro* [39]. Co_3_O_4_ NPs were synthesized having spherical morphologies with a diameter size of 50–60 nm, using *Calotropis gigantea* leaf extracts that indicate the presence of triterpenoids, flavonoids (polyphenols), steroids, cardenolides, and alkaloids [40]. *Hibiscus cannabinus* leaf extract was used to synthesize crystalline CoNPs having a diameter of 20.8 nm. These CoNPs were characterized by UV-vis, XRD, and Fourier-transform infrared spectroscopy (FTIR). Authors reported that the green-synthesized NPs have strong activities against *Bacillus subtilis* and *Escherichia coli* [41]. Onwudiwe et al. (2020) reported the bio-mediated synthesis of Co_3_O_4_ NPs using the fruit extract of *Litchi chinensis.* The characterizations of synthesized NPs were confirmed by XRD, SEM, transmission electron microscopy (TEM), and FTIR. SEM analysis revealed that the bioinspired NPs had elongate rod-like morphology. FTIR analysis was also carried out to detect the functional group of various phytochemicals that are involved in the oxidation/reduction and hence the formation of cobalt oxide NPs. The FTIR analysis confirmed the presence of phenolic and carboxylic functional groups, indicating that these groups are responsible for the formation of cobalt oxide NPs [42]. *Sechium edule* and *Helianthus annuus* fruits’ extracts [34,44] and *Piper nigrum* seeds [45] have also been reported for the synthesis of Co_3_O_4_ NPs. *Vitis vinifera* whole plant extracts were used for the synthesis of Co_3_O_4_ NPs having a pure single crystalline structure with a size of 10–20 nm in diameter. The TEM and FTIR analysis indicated that the plant extracts play an important role in the stabilization and reduction of nanorods via different types of organic compounds present in the plant extracts [46]. Latex derived from *Calotropis procera* was used to synthesize Co_3_O_4_ NPs with spherical morphologies and average size of 10 nm diameter. These NPs showed only a low toxicity at a very high concentration, proving that they are safe and may be used for different applications in various fields including the field of medicines [47]. Other than aforementioned, different parts of various plants, used for the synthesis of Co and Co_3_O_4_ NPs are shown in Table 1 and Figure 3.

### 3.2 Synthesis of Co and Co_3_O_4_ NPs using microorganisms

#### 3.2.1 Bacteria-mediated synthesis

Similar to other synthesis routes such as those of plants and other microorganisms, bacteria also have an intrinsic ability to synthesize NPs of different sizes and morphologies. To date, different types of bacterial strains have been used to synthesize Co and Co_3_O_4_ NPs [33,72,74]. This approach also has some challenges compared to other methods that are yet to be solved, like the synthesis of complex materials with the desired phase, a full understanding of the synthesis mechanism at the molecular level to achieve better control over shape and size, and scaling up in order to obtain large amounts of nanomaterials [73]. Chemical methods are already advanced to provide greater control on the shape and size of nanomaterials, but this approach is eco-friendly and limits the drawbacks of the chemical synthesis [73]. However, lengthy procedures and contamination of cultures are the limitations of this approach. Different bacterial species have been used for the synthesis of cobalt and cobalt oxide NPs.

A gram-positive bacterium *Bacillus thuringiensis* was used to synthesize Co NPs having face-cubic morphology with an average size of 85.3 nm diameter. These green-synthesized NPs were investigated against malarial and dengue vectors (*Aedes aegypti* and *Anopheles subpictus*) and showed potential activities against these vectors [75]. Co_3_O_4_ NPs synthesized using bacterial strains *Micrococcus lylae* and *B. subtilis* have globular and rod shape morphology, respectively [74]. Gram-negative bacterium *Proteus mirabilis* was used to synthesize CoNPs with an average size of 57 nm and quasi-spherical morphology. The authors evaluated the antibacterial activity of these NPs, and the result showed that these NPs were promising antimicrobial potential against *Salmonella typhi*, *E. coli*, *Clostridium perfringens*, *Staphylococcus aureus*, and *Bacillus cereus* [76]. Table 2 provides some examples of bacterial-mediated synthesized Co and Co_3_O_4_ NPs.

**Table 2:** Bacterial-mediated synthesis of Co and Co_3_O_4_ NPs

Bacterial strain	Gram+/Gram−	NPs	Characterization	Size (nm)	Shape	References
*Bacillus pasteurii*	Gram+	Cobalt oxide	XRD, SEM, FE SEM, TEM, and HR-TEM	10–31	Irregular	[33]
*Bacillus subtilis*	Gram+	Cobalt oxide	XRD, SEM, FE SEM, TEM, and HR-TEM	6.6	Hollow rod	[72]
*Brevibacterium casei*	Gram+	Cobalt oxide	XRD, SEM, FE SEM, TEM, and HR-TEM	6	Quasi-spherical	[73]
*Micrococcus lylae*	Gram+	Cobalt oxide	XRD, SEM, FE SEM, TEM, and HR-TEM	8	Flower like	[77]
*B. subtilis*	Gram+	Cobalt oxide	FESE, M ELS-Z2, FESEM, XPS, and EPMA	3–5	Rods	[78]
*M. lylae*	Gram+	Cobalt oxide	XRD, TGA, FE-SEM, TEM, EDS, SAED, and HAADF-STEM	40	Spherical	[79]
*M. lylae*	Gram+	Cobalt	Zetasizer Nano system and FESEM	356 ± 55	Globular	[74]
*B. subtilis*	Gram+	Cobalt	Zetasizer Nano system and FESEM	NA	Rod shape	[74]
*Escherichia coli*	Gram−	Cobalt	Zetasizer Nano system and FESEM	473 ± 54	Rod shape	[74]
*Paracoccus* sp.	Gram−	Cobalt	Zetasizer Nano system and FESEM	NA	Biconcave	[74]
*Haloarcula vallismortis*	Gram−	Cobalt	Zetasizer Nano system and FESEM	NA	Globular	[74]
*Proteus mirabilis*	Gram−	Combine Cobalt	UV-vis, EDX, XRD, TEM, DLS, and PDI	57	Quasi-spherical	[76]
*Bacillus thuringiensis*	Gram+	Cobalt	XRD, TEM, FESEM, FTIR, and EDX	85.3	Face-cubic	[75]

Abbreviations: NPs, nanoparticles; NA, not available; SAED, selected area electron Diffraction; UV-vis, Ultraviolet visible spectroscopy; XPS, X-ray photoelectron spectroscopy; TEM, transmission electron microscopy; XRD, X-ray diffraction; HR-TEM, high-resolution transmission electron microscopy; EDX, energy-dispersive X-ray(spectroscopy); FTIR, Fourier-transform infrared spectroscopy; TGA, thermo gravimetric analysis; ATR-FTIR, attenuated total reflectance-Fourier transform infrared spectroscopy; FESEM, field emission scanning electron microscopy; EPMA, electron probe microanalyzer; HAAD-STEM, high-angle annular dark field scanning TEM; EDS, energy-dispersive X-ray spectroscopy.

#### 3.2.2 Fungi-mediated synthesis of Co_3_O_4_ NPs

The fungi-mediated approach exhibits unique advantages, as the growth process of fungi is easy to handle and isolate, with the large amount of biomass and high yield of proteins [34]. The entophytic fungi also secrete large amounts of bioactive substances that are necessary for the synthesis of NPs in the presence of precursor substance [34,80]. During the fabrication of NPs from the precursor solution, biomass (fungi) along with the supernatant act as a reduction medium [34,81].

Using fungi, NPs can be synthesized by two pathways: intracellular and extracellular synthesis. The extracellular approach is more commonly used because of the facile separation, high mass harvesting, and easy culturing, which consequently leads to the scaling up of the process at the commercial level [82]. Fungi can also tolerate agitation, flow pressure, and other conditions in the bioreactor for commercial production. Filamentous fungi are highly resistant toward metals as compared to bacteria [83,84]. Different fungal and yeast strains have been used to synthesize cobalt oxide NPs. *Aspergillus nidulans* was used to synthesize spherical shape Co_3_O_4_ NPs with an average size of 20.29 nm diameter [34]. Some examples of yeast and fungi-mediated synthesis of Co_3_O_4_ NPs are presented in Table 3.

**Table 3:** Fungal-mediated synthesis of Co and Co_3_O_4_ NPs

Fungal species used	NPs	Characterization	Size (nm)	Shape	References
*Aspergillus nidulans*	Cobalt oxide	XRD, TEM, FTIR, and EDX	20.29	Spherical	[34]
*Aspergillus brasiliensis*	Cobalt oxide	DLS, EDX, XRD, FT-IR, HR-TEM, and FESEM	20–27	Quasi-spherical	[83]
*Nothapodytes foetida*	Cobalt oxide	UV-vis	N/A	H/A	[85]
Yeast	Cobalt oxide	SEM, XRD, and FT-IR	24	Hollow spheres	[86]

Abbreviations: NPs, nanoparticles; NA, not available; UV-vis, ultraviolet visible spectroscopy; XPS, X-ray photoelectron spectroscopy; TEM, transmission electron microscopy; XRD, X-ray diffraction; HR-TEM, high-resolution transmission electron microscopy; EDX, energy-dispersive X-ray(spectroscopy); FTIR, Fourier-transform infrared spectroscopy; VSM, vibrating-sample magnetometer; FESEM, field emission scanning electron microscopy.

### 3.3 Algal-mediated approach for the synthesis

Algae are aquatic microorganisms that are used to a great extent for the synthesis of NPs. Algae are also called bionanofactories because they fabricate nanomaterials with high stability, are easy to handle, and do not need cell maintenance [87]. Algae are the key origin of bioactive metabolites that are involved in the fabrication of NPs [88,89]. They contain many bioactive metabolites such as proteins, polysaccharides, and various types of other phytochemicals that are comprised of amino, hydroxyl, and carboxyl functional groups, which are responsible for the fabrication of NPs [87,90]. Algae differ in size and can vary from microalgae to macroalgae [87,88,89,90]. A green-type macroalgae *Enteromorpha flexuosa* was used to synthesize cobalt–ferrite NPs with an average size of 5–15 nm. These bioinspired cobalt ferrite NPs were characterized by various advanced techniques such as XRD, SEM, TEM, energy-dispersive X-ray spectroscopy (EDS), and XPS [91]. Similarly, cobalt tungstate (CoWO_4_) NPs synthesized from red seaweeds (*Rhodophyta*) using agar-agar have also been reported [92].

### 3.4 Biological product-mediated synthesis of Co and Co_3_O_4_ NPs

The plants and microorganisms are widely used for the fabrication of NPs. Besides these, the biological derivatives are used to synthesize NPs as well. They also play a significant role in the stabilization and reduction of nanomaterials [93]. Co_3_O_4_ nanocrystals were synthesized from freshly extracted ovalbumin with 8–17 nm diameter confirmed by EPR [94]. Sun et al. (2019) also reported the synthesis of Co_3_O_4_ NPs using egg-albumin. Hosein et al. (2004) reported the synthesis of Co_3_O_4_ NPs with a uniform shape and size using ferritin [95]. Starch has also been exploited for the synthesis of carbon-encapsulated Co NPs with an average size of 20–35 nm diameter and confirmed by the nanostructure by SEM, TEM, and XRD [96]. Similarly, peptide TLVNN (threonine–leucine–valine–asparagine–asparagine) was also used as a capping agent for *in situ* syntheses of CoNPs for bioapplications [97].

## 4 Biological activities of Co and Co_3_O_4_ NPs

### 4.1 Antibacterial activity

Currently, throughout the globe, the emergence of bacterial resistance to the available antibiotics is a major health concern. Therefore, there is a need for the antibiotic agent that can kill pathogenic bacteria which show resistance to the available drugs [98]. The NPs have small size with high surface area compared to the bigger molecules and therefore possess strong antibacterial activities. The NPs have dose-dependent membrane permeation and inhibit the synthesis of bacterial proteins by disturbing the cell membrane [32,99]. Different metallic NPs such as gold, iron, silver, and metal oxide NPs such as iron oxide, cobalt oxide, and copper oxide showed significant antibacterial activities. The AgNPs are the main interest not only in biomedical industries but also in food industries because of its potential antimicrobial behavior [114]. Cobalt and cobalt oxide NPs also possess potential antibacterial activity. Varaprasad et al. (2017) measured the antibacterial activity of biogenic CoNPs against human pathogenic bacteria, and the results concluded that CoNPs have strong antibacterial activities compared to standard antibiotic drug ciprofloxacin [100]. Eltarahony et al. (2018) reported the green synthesis of combined CoNPs using gram-negative bacteria *P. mirabilis*. The characterization of NPs was confirmed by UV-vis, EDX, XRD, TEM, dynamic light scattering (DLS), and poly dispersity index (PDI). The antibacterial activity was observed using a well diffusion assay against *S. typhi*, *E. coli*, *C. perfringens*, *S. aureus*, and *Enterococcus faecalis* [76]. The result showed that the NPs have promising biocide efficiency against these bacteria [76]. Omran et al. (2019) synthesized quasi-spherical shape cobalt oxide NPs with an average size of 20–27 nm diameter using a fungal specie *Aspergillus brasiliensis*. The first-time mycosynthesized cobalt oxide NPs showed considerable activity against different bacteria [83]. Biogenic synthesis of CoNPs using *Celosia argentea* whole plant extract has been reported and studied for their antibacterial activities using the disk diffusion method. The synthesized NPs showed remarkable antibacterial activities against *B. subtilis* and *E. coli* [32]. Green-mediated cobalt oxide NPs have been synthesized using *Hibiscus rosa-sinensis* flower extract and their antibacterial activity was measured. These green-synthesized NPs showed promising activities against *E. coli*, *Streptococcus mutans*, *S. aureus*, and *Klebsiella pneumonia* [101]. Mainly two aspects have been suggested. First, in cobalt oxide NPs, the different positive states of cobalt ions, i.e., Co^2+^ and Co^3+^ interact with the parts of the bacterial cell that have a negative charge and cause the death of the bacterial cell. Second, there may be the excitement of electrons on the surface of cobalt oxide because of light irradiation in the conduction and valence band. In the conduction band, there is the formation of superoxide radical anion because of the reaction of excited electrons and oxygen molecules. Finally, the formation of hydrogen peroxide, which is a strong oxidant, occurs. On the surface of NPs, the reaction of water and superoxide radical anion destroys the bacterial cell. Therefore, Cobalt oxide nanoparticles can be a potent antibacterial agent at a minimal level of concentration [6,102]. The antibacterial activities of cobalt and cobalt oxide NPs synthesized from different green routes are presented in Table 4.

**Table 4:** Antibacterial activities of green-synthesized cobalt and cobalt oxide NPs

Biological entity	NPs	Test microorganisms	Method	References
*Celosia argentea*	Cobalt	*Bacillus subtilis* and *Escherichia coli*	Disk diffusion	[32]
*Ziziphora clinopodioides* Lam	Cobalt	*Salmonella typhimurium*, *E. coli*, *Streptococcus pneumonia*, *Pseudomonas aeruginosa*, *Staphylococcus aureus*, and *B. subtilis*	Disk diffusion	[37]
*Raphanus sativus* var. longipinnatus	Cobalt	*Pseudomonas putida* and *Klebsiella pneumonia*	Disk diffusion	[39]
*Hibiscus cannabinus*	Cobalt	*B. subtilis* and *E. coli*	Agar well diffusion	[41]
*Vitis vinifera*	Cobalt oxide	*P. aeruginosa*, *E. coli*, *S. aureus*, and *B. subtilis*	Disk diffusion	[46]
*Calotropis procera*	Cobalt oxide	*E. coli*, *Pseudomonas* sp., *Alcaligenes* sp., and *Enterococcus* sp.	Disc diffusion	[47]
*Moringa oleifera*	Cobalt	*E. coli* and *S. aureus*	Agar well diffusion	[51]
*Sechium edule*	Bimetallic cobalt	*B. subtilis* and *E. coli*	Disk diffusion	[60]
*Populus ciliata*	Cobalt oxide	*Bacillus licheniformis*, *B. subtilis*, *K. pneumonia*, and *E. coli*	Well diffusion	[63]
*Sageretia thea*	Cobalt oxide	*P. aeruginosa*, *E. coli*, *K. pneumonia*, *Staphylococcus epidermis*, *S. aureus*, and *B. subtilis*	Disc diffusion	[65]
*Chromolaena odorata*	Cobalt	*E. coli*, *K. pneumonia*, *S. aureus*, and *Streptococcus pyogenes*	Agar well diffusion	[69]
*Catharanthus roseus/Periwinkle*	Cobalt	*B. subtilis* and *E. coli*	Disc diffusion	[70]
*Sesbania sesban*	Cobalt oxide	*S. aureus*	Disk diffusion	[71]
*Proteus mirabilis*	Cobalt	*P. aeruginosa*, *Salmonella typhi*, *E. coli*, *Clostridium perfringens*, *Enterococcus faecalis*, *Bacillus cereus*, and *S. aureus*	Well diffusion	[76]
*Aspergillus brasiliensis*	Cobalt oxide	*B. subtilis*, *S. aureus*, *P. aeruginosa*, and *E. coli*	Agar well diffusion	[83]

Abbreviation: NPs, nanoparticles.

### 4.2 Antifungal activity

The resistance of bacteria and fungi to the available antibiotics and drugs is at an alarming rate. Therefore, there is a need for strong antifungal agents that can destroy fungi that are resistant to the drugs available [19]. Cobalt and cobalt oxide NPs have various biomedical applications because of different properties including antifungal property. Hou et al. (2020) measured the antifungal properties of green-synthesized CoNPs, and the result showed that the CoNPs have strong antifungal activities against *Candida krusei*, *Candida guilliermondii*, *Candida glabrata*, and *Candida albicans* [37]. Similarly, cobalt oxide NPs synthesis using flower extract of *H. rosa-sinensis* and their antifungal activities has been reported. The result showed that the synthesized NPs have strong activities against *Aspergillus flavus* and *A. niger* [62].

### 4.3 Larvicidal activities of CoNPs

For tropical and subtropical countries, the vector and vector-borne diseases have become a big trouble for public health [103]. For the human vector-borne infectious disease, the microorganisms (parasite, bacterium, or virus) and the vectors (mosquito, fly, or tick) are the crucial elements [104]. Mosquitos are the vector of infectious diseases such as dengue, malaria, fever, and yellow fever [104]. The mosquitoes affect humans as well as domestic animals throughout the globe. The biocontrol method is being considered because of high resistance to the chemical insecticides and no new approaches to control the mosquitos. In this approach, different microbes, e.g., *B. thuringiensis*, have been used in regards to their toxicity to the target vector [75,105,106].

*A. subpictus* and *A. aegypti* are the vectors that cause malaria and dengue, respectively, and are the main species of medical interest. In more than 100 countries, about 2.5 billion people are at risk of infection with dengue. Fifty million people are affected worldwide with more than 24,000 deaths per year. There is a need for development of new agents to control these vectors [75]. CoNPs have also been tested against malarial *A. subpictus* and *A. aegypti*. Marimuthu et al. (2013) tested the bacterial-mediated synthesized CoNPs using *B. thuringiensis* against malarial and dengue vectors *in vitro*. The results showed that the green-synthesized CoNPs have promising activities that exceed those of biocontrol agent (*B. thuringiensis*) against these vectors [75]. Therefore, it may possible to use the CoNPs in drug formulation against these parasites.

### 4.4 Antileishmanial activity

The World Health Organization considered the leishmaniasis as one of the uncontrolled, emerging, and neglected diseases with the second-highest prevalence rate, with malaria placing itself at the topmost among the parasitic diseases [107]. Leishmaniasis is caused by leishmania parasites that exist in two forms, promastigote (motile) and amastigote (non-motile) [108]. Leishmaniasis occurs mainly in two clinical forms: visceral leishmaniasis (also called *kala-azar*), which is the most severe form and - if left untreated - may lead to death, and cutaneous leishmaniasis. It is predicted that about 0.5 million cases of visceral leishmaniasis and 1.5 million cases of cutaneous leishmaniasis occur throughout the globe per year [109]. This deadly disease is endemic in 98 countries of the world. Currently, no vaccines are available against leishmaniasis. The only option available are the treatment drugs and none of the available drugs is ideal because of their cost, duration of therapy, severe side effects, high toxicity, and - most importantly - the resistance of leishmania parasites to the available treatment [107]. Therefore, there is an immediate need to develop an effective antileishmanial approach to combat this deadly infection.

Talha et al. (2017) synthesized cobalt oxide NPs using the extract of *Sageretia thea* (Osbeck.), a medicinal plant. The characterizations were confirmed by XRD, FTIR, EDS, selected area electron diffraction, high-resolution scanning electron microscopy, and high-resolution transmission electron microscopy. For the first time the antileishmanial activity of the green-synthesized NPs was evaluated using MTT cytotoxic assay. The results showed that the antileishmanial response was dose dependent, and the amastigote parasites were most susceptible as compared to the promastigote. Therefore, cobalt oxide NPs may be one of the possible options in nanomedicine to treat leishmania at any stage of the life cycle [50].

### 4.5 Antioxidant activity

Oxidative metabolism is a key process for the survival of cells. However, this process has some side effects as they produce free radicals and reactive oxygen species. When these free radicals are produced in the body in the excess amount they can inundate the enzymes such as catalases, peroxidase, and superoxide dismutase and lead to lethal cellular effects by oxidizing cellular proteins, membrane lipids, DNA enzymes, and influence signaling pathways of the cell leading to termination of cellular respiration [110]. Oxidation affects food as well, which is one of the main causes of chemical spoilage that affects flavor, texture, nutritional value, and safety of food. Different types of natural and synthetic antioxidants are available to limit the side effects of oxidation [110,111].

NPs also have strong antioxidant activities. Plant-mediated cobalt oxide NPs having cubic shape morphologies with an average size of 20.03 nm diameter have been prepared using the leaf extract of *S. thea*. The antioxidant assays, free radical scavenging, total antioxidant capacity, and total reducing power have been evaluated. The biogenic cobalt oxide NPs showed superior radical scavenging potential and an average total antioxidant capacity and total reducing power [50]. Shahzadi et al. (2019) also observed radical scavenging activity of bioinspired CoNPs and reported that the scavenging power and antioxidant activity are dose dependent: the increase of activity leads to the increase in the concentration of CoNPs [32]. CoNPs using the leaf extract of *Ziziphora clinopodioides* Lam has been synthesized and the antioxidant activities were evaluated. The green-synthesized NPs showed impressive results and have good DPPH free radical scavenging activity [37]. Similarly, DPPH radical scavenging activity of cobalt oxide NPs synthesized from the *Sesbania sesban* extract has been reported to have minimum activities compared to silver and copper oxide NPs [71].

### 4.6 Cytotoxic activity

Cytotoxicity assay is a test for the characterization and evaluation of potentially harmful and toxic effects of biomolecules or any other materials on the living organisms/cell cultures. Various types of molecules, plant extracts, and NPs have been used to check the cytotoxic effect [112]. The green-synthesized CoNPs were used in different concentrations to investigate and determine the cytotoxicity of human umbilical vein endothelial cells (HUVECs) *in vitro*. The cells treated with various concentrations of CoNPs were examined by the MTT test for 48 h. The absorbance rate was determined at 570 nm, which indicated good viability on (HUVECs) cell lines even up to 1,000 mg/mL of CoNPs, and the absence of such type of toxicity of CoNPs has many safe applications in nanomedicines [37]. Padigya et al. (2016) observed the cytotoxic effect of the biogenic CoNPs on HeLa cell lines. They reported that CoNPs showed potential cytotoxic effects against HeLa cancer cell lines [39]. The green-synthesized cobalt oxide NPs were also investigated for cytotoxicity through brine shrimp cytotoxicity assay. Cytotoxicity of the Co_3_O_4_ NPs was confirmed by their dose-dependent response, whereas the median lethal concentration was calculated as 19.18 μg/mL [50].

### 4.7 Hemolytic activities

Hemolysis is the release of hemoglobin in the blood because of the disruption of erythrocyte membranes that may lead to jaundice or anemia. That is why it is very important to check the hemolytic activity of any newly synthesized preparate that can be used for pharmacological purposes [113]. Shahzadi et al. (2019) evaluated the hemolytic activity of green-synthesized CoNPs using the plant extract of *C. argentea*. The results showed that the biosynthesized CoNPs have less hemolytic activity (2.95%) compared to positive control triton-X-100 which has 95.25% toxicity, whereas the negative taken has 1.02% toxicity [32]. Zaib et al. (2019) fabricated CoNPs using the *Catharanthus roseus* extract and evaluated their hemolytic activities. They reported that the hemolysis rate of CoNPs is approximately 1.53%, with the negative and positive control rates of 1.02% and 95.28%, respectively [70]. Therefore, CoNPs can be used in drug formation because of their safety, cost-effectiveness, and nontoxic nature. Cobalt oxide NPs were prepared using *S. thea* extract and evaluated in regards to their biocompatibility and various biological applications. The result presented that the median lethal dose concentration was observed as >58.55 µg/mL and 200 µg/mL for macrophages and RBCs, respectively [50]. For that reason the biosynthesized cobalt oxide NPs might be used at low concentrations for drug formulations.

### 4.8 Other biological applications

Apart from antimicrobial, antioxidant, antileishmanial, cytotoxic, hemolytic, and larvicidal activities, Co and Co_3_O_4_ NPs have various biological and medical applications. After cardiovascular diseases, cancer is the second main cause of human dysphoria [19]. CoNPs showed promising anticancer activities. Kgosiemang et al. (2020) synthesized cobalt oxide NPs using the *Euphorbia tirucalli* extract and investigated the anti-proliferative activity using MTT assay against MCF-7 breast cancer cell lines. The result showed that the biosynthesized cobalt oxide NPs exhibit promising activities against MCF-7 breast cancer cell lines [53]. The cutaneous wound healing potential of green-synthesized CoNPs has been investigated and it was reported that the ointment of CoNPs has great potential in cutaneous wound healing [37]. The catalytic activity, enzyme inhibition, antidiabetic activity, and anticholinergic activity of biogenic cobalt and cobalt oxide NPs have been reported [37,50].

## 5 Conclusion

The green-synthesized cobalt and cobalt oxide NPs have various biological and biomedical applications. Traditionally, NPs are synthesized by either physical or chemical methods, which leads not only to environmental toxicity but also to costly and energy-intensive labor. Cobalt and cobalt oxide NPs are synthesized by a green route using the extracts of different plants/parts of plants, microorganisms, and other biological molecules such as gelatin, oleic acid, and starch. The biomediated cobalt and cobalt oxide NPs are environmentally friendly, facile in terms of synthesis, cost effective, and biocompatible.
